# Species distribution and antibiotic susceptibility profile of bacterial uropathogens among patients complaining urinary tract infections

**DOI:** 10.1186/s12879-017-2743-8

**Published:** 2017-09-29

**Authors:** Adane Bitew, Tamirat Molalign, Meseret Chanie

**Affiliations:** 1 Department of Medical Laboratory Sciences, College of Health Sciences, Addis Ababa University, Addis Ababa, Ethiopia; 2 Department of Medical Laboratory, St. Peter Tuberculosis Specialized Hospital, Addis Ababa, Ethiopia; 3 Arsho Advanced Medical Laboratory Private Limited Company, Addis Ababa, Ethiopia

**Keywords:** UTIs, Drug resistance, Species distribution, Ethiopia

## Abstract

**Background:**

Urinary tract infection is the second most common type of infection and the problem is further compounded by the emergence of drug resistance in bacterial uropathogens. The aim of this study was to determine the spectrum of bacterial uropathogens and their drug resistant pattern.

**Methods:**

A single institutional cross-sectional study was carried out at Arsho Advanced Medical laboratory from September 2015 to May 2016. A total of 712 urine samples were collected, inoculated onto primary isolation culture media, incubated at 37 °C for 18–24 h, and significant bacteriuria was determined. Identification and the antimicrobial susceptibility testing of bacteria were determined by using the automated VITEK 2 compact system.

**Results:**

Out of 712 urine samples processed, 256 (36%) yielded significant bacteriuria of which 208 (81.25%**)** were obtained from female and 48 (18.75%) from male patients. Age group of 25–44 were more affected with the infection. Of 256 bacterial isolates recovered, *Escherichia coli,* was the dominant bacterium. Ampicillin and trimethoprim/sulfamethoxazole were the least effective drugs while piperacillin/tazobactam was the most effective drug against Gram-negative bacteria. Erythromycin was the least effective drug while vancomycin was the most active drug against Gram-positive bacteria.

**Conclusions:**

Observation of many bacterial species causing UTI in this study warrants, a continuous epidemiological survey of UTI in health institutions across the country. High level of drug resistance to the commonly prescribed drugs necessitates a search for other options.

## Background

Urinary tract infection (UTI) is an infection of the bladder (cystitis) or the kidneys (pyelonephritis). It is the second most common type of infection accounting for about 8.1 million visits to health care providers each year [1]. Women are more are more susceptible to UTI than men. Over 50% of all women will experience at least one UTI during their life-time, with 20–30% experiencing recurrent UTI [2, 3].

Urinary tract infection is a morbid disease in terms of loss of working days and treatment cost [4]. In the United States alone, UTI has been reported to cause > 6 million outpatient visits [5] and 479,000 hospitalizations annually [6]. Furthermore, the annual treatment cost of UTI in this part of the world has been estimated to be greater than 2.47 billion USD [2]. They are also important cause of sepsis resulting in high mortality rates [7].

Infants, pregnant women, patients with spinal cord injuries, diabetes, multiple sclerosis, acquired immunodeficiency disease syndrome or underlying urologic abnormalities are subjects that are at increased risk for UTI. In addition, catheter-associated UTI is the most common nosocomial infection [7].

Many previous studies have shown that *E. coli* is the most common etiological agent of UTI in both hospital and community acquired infections. Hospital acquired UTI has also been characteristically associated with a higher prevalence of enterococci and Coagulase- Negative Staphylococci [8–13]. In addition, *Klebsiella pneumonia, Streptococcus agalactiae*, *Proteus mirabilis,* viridans streptococci, *Klebsiella oxytoca*, *Pseudomonas aeruginosa*, *Citrobacter freundii*, *Enterobacter cloacae,* and *Staphylococcus aureus* have been identified as etiologic agents of UTI [7].

Due to the rapidly evolving adaptive strategies of bacteria, the etiology of UTI and antibiotic resistance profile of bacterial uropathogens have changed considerably over the past years, both in community and nosocomial infections [13]. Many studies conducted from the USA and Europe have revealed increasing antibiotic resistance among uropathogenic *E. coli* to ampicillin, trimethoprim, and sulfonamides [9, 10, 12]. Apparent shift in the etiological agents of urinary tract infection and associated problem of antibiotic resistance amongst bacterial uropathogens from time to time and from one institution to another have initiated health institution to carry out continuous evaluation of UTI from the view point of their spectrum and drug susceptibility testing.

Accurate identification of bacterial uropathogens and determining their drug susceptibility pattern are critical for efficient management of patients with UTI. They are also associated with significant clinical and financial benefits, via the reduction of mortality rates and overall hospitalization costs [14]. In view of this, identification and antimicrobial susceptibility testing of clinical isolates by means of fully automated systems have become a common practice in many laboratories. The VITEK 2 compact system is a new automated system designed to provide accurate identification and susceptibility testing results for most clinical isolates of both Gram-positive and Gram-negative bacteria. Apart from accurate identification and susceptibility testing shortened turnaround times, improved specimen handling, enhanced quality control, reproducibility and the ability to track results are further advantages of the system [15].

Unfortunately, in Ethiopian health care providing institutions, identification and drug susceptibility profile of bacterial uropathognes have been carried by conventional methods that appeared to be inferior to the fully automated systems. Against this background, the present study was designed to determine the spectrum of bacterial uropathogens and their antimicrobial susceptibility profile by employing the VITEK 2 compact system among patients referred to Arsho advanced medical laboratory private limited company with a complain of UTI.

## Methods

### Study site, period and socio-demographic data

The present study was a single institutional cross-sectional study carried out at Arsho Advanced Medical laboratory, Addis Ababa, Ethiopia from June 2015 to May 2016. Willingness to participate in the study, presumptive diagnosis of urinary tract infection and no history of antibacterial therapy within 2 weeks prior to their attendance were the inclusion criteria. The requisition form filled up by physicians was used as standard proforma to document socio-demographic information of study subjects. Age groups of patients were classified following WHO guideline [16].

### Sample collection and inoculation of primary isolation culture media

Clean-catch midstream urine was collected from patients complaining of UTI; referred from different health institutions in the city with sterile wide-mouthed urine cup. Urine samples were inoculated onto Blood Agar base (Oxoid, Basingstoke, Hampaire, UK) to which 10% sheep blood is incorporated and Cysteine Lactose Electrolyte Deficient medium (Oxoid, Basingstoke, Hampaire, UK) using a calibrated loop with a capacity of 1 μl in safety cabinet. All inoculated plates were incubated at 37 °C for 18–24 h aerobically and the number of colonies was counted. Colony counts yielding bacterial growth of *>*10^5^/ml of urine (≥100,000 colonies) were regarded as significant for bacteriuria. Urine samples yielded three and more bacterial species were not considered for further investigation. Pure isolates of bacterial pathogen were preliminary characterized by colony morphology, Gram-stain, and catalase test before inoculating them into AST-GN72 and AST-GP71 cards.

### Inoculum size determination

Quality control bacteria and pure cultures of bacterial isolates were suspended in 3 ml of sterile saline (aqueous 0.45 to 0.50% NaCl, pH 4.5 to 7.0) in a 12 × 75 mm clear plastic (polystyrene) test tube to achieve a turbidity equivalent to that of a McFarland 0.50 standard (range, 0.50 to 0.63), as measured by the DensiChek (bioMe’rieux) turbidity meter. These suspensions were used for the inoculation of GN72 and GP71 identification cards while AST cards were inoculated after bacterial suspensions were further diluted following the instruction of the manufacture.

### Identification and determination of antimicrobial susceptibility

Species identification and antimicrobial susceptibility testing of Gram-positive and Gram-negative bacteria were determined with the automated VITEK 2 compact system (bioMérieux, France) using AST, GN72 and GP71 cards. The VITEK 2 compact system (bioMe’rieux) is an integrated modular system that consists of a filling-sealer unit, a reader-incubator, a computer control module, a data terminal, and a multicopy printer. The system detects bacterial growth and metabolic changes in the microwells of thin plastic cards using a fluorescence-based technology.

AST-GN72 cards were used for the identification and susceptibility testing of none-spore-forming, fermenting, and non-fermenting Gram-negative bacilli while the AST-GP71 cards were used for the automated identification and susceptibility testing of non-spore-forming Gram-positive bacteria. The cards were automatically filled by a vacuum device and were automatically sealed and subjected to a kinetic fluorescence measurement in accordance with the manufacturer’s instructions. Brief, identification cards were inoculated with quality control bacteria and pure cultures of bacterial isolate suspensions using an integrated vacuum apparatus. A test tube containing the bacterial suspension was placed into a special rack (cassette) and the identification card was placed in the neighboring slot while inserting the transfer tube into the corresponding suspension tube. The filled cassette was inserted manually into the VITEK 2 compact reader-incubator module. After the vacuum was applied and air was re-introduced into the station, the bacterial suspension was forced through the transfer tube into micro-channels that fill all the test wells and inoculated cards were automatically sealed prior to loading into the carousel incubator. All card types were incubated automatically incubated 35.5 ± 1.0 °C. Each card was removed from the carousel incubator once every 15 min, transported to the optical system for reaction readings, and then returned to the incubator until the next read time. Data were collected at 15-min intervals during the entire incubation period and final identification results were obtained in approximately 18 h or less. All cards used were automatically discarded in a waste container.

AST-GN72 cards consists of 64 biochemical method and substrates for identification and a panel of 19 antibiotics for drug susceptibility testing. Antibiotics with their different concentration used for the determination of drug susceptibility profile of Gram-negative bacteria in this investigation were: ampicillin (4,8,32), amoxicilin/clavulanic acid (4/2,16/8,32/16), cefalotin (2,8,32), cefazolin (4, 16, 64), cefepime (2,8,16,32), cefoxitin (8,16,32), cefpodoxime (0.5, 1, 4), ceftazidime (1,2,8,32), ceftriaxone (1,2,8,32), cefuroxime (2,8,32), ciprofloxacin (0.5,2,4), gentamicin (4,16,32), levofloxacin (0.25,0.5,2,8), nitrofurantoin (16,32,64), piperacillin/tazobactam (2/4,8/4,24/4,32/4,32/8), tetracycline (2,4,8), tobramycin (8,16,64), trimethoprim/sulfamethoxazole (1/19,4/76,16/304).

Similarly, the AST-GP71 card consists of an array of biochemical tests for species characterization and antibiotics for drug susceptibility testing of Gram-positive bacteria. Antibiotics with their different concentration used for the determination of drug susceptibility pattern of Gram-positive bacteria in this investigation were:- cefoxitin screen (6), ciprofloxacin (1, 2, 4), clindamycin(0.5,1,2), daptomycin (0.5, 1, 2, 4, 16), erythromycin (0.25,0.5,2), gentamicin (8,16,64), inducible clindamycin resistance (CM 0.5, CM/E 0.25/0.5), levofloxacin (0.25,2,8), linezolid (0.5,2,8), minocycline (0.12,0.5,1), moxifloxacin (0.25,2,8), nitrofurantoin (16,32,64), oxacillin (0.5,1,2), quinupristin/dalfopristin (0.25,0.5,2), rifampicin (0.25,0.5,2), tetracycline (0.5,1,2), tigecycline (0.25,0.5,1), trimethoprim/sulfamethoxazole (2/38,8/152,16/304), and vancomycin (1,2,4,8,16).

### Quality control

For quality control of susceptibility tests *E. coli* ATCC 25922, *P. aeruginosa* ATCC 27853, *S. aureus* ATCC 25923 and *E. faecalis* ATCC929212 strains were used.

### Ethical clearance

All ethical considerations and obligations were duly addressed and the study was conducted after the approval of the Department Research and Ethical Review Committee (DRERC) of the Department of Medical Laboratory Sciences, College of Health Sciences, and Addis Ababa University. Informed written consent was obtained from participants before data collection. The respondent was given the right to refuse to take part in the study and to withdraw at any time during the study period. All the information obtained from the study subjects were coded to maintain confidentially. When the participants were found to be positive for bacterial pathogen, they were informed by the hospital clinician and received proper treatment. Assent form was completed and signed by family member and/or adult guardian for participants under the age of 16 years.

## Results

Of a total of 712 urine samples processed during the study period, 519 (72.9%) were collected from female patients and 193 (27.1%) from male patients. Two hundred fifty six (36%) urine samples yielded significant bacteriuria of which 208 (81.2%**)** were obtained from female patients and 48 (18.8%) from male patients. Cases of 75% UTI were recorded among young and middle age patients with an age group of 15–64 years. Pediatric patients (0–14 years) and elderly patients (≥65 years) accounted for 11.3 and 13.7% of the total number of UTI, respectively (Table 1). Urinary tract infection was the highest (43.8%) in patients of age group 25–44 followed by age groups of 45–64 (20%).

**Table 1:** Gender and age distribution of study participants

Variable	Categories	Samples collected *n* = 712	Sample s collected *n* = 712	
			UTI yes = 256	UTI no = 456
Gender	Female	519 (72.9)	208(81.2)	311(68.2)
	Male	193 (27.1)	48(18.8)	145 (31.8)
	Total	712 (100)	256(100)	456 (100)
Age group	<1	28 (3.9)	18(7.0)	10 (2.2)
	1–14	55 (7.7)	11(4.3)	44 (9.6)
	15–24	74 (10.4)	29(11.3)	45(9.9)
	25–44	330 (46.3)	112(43.8)	218(47.8)
	45–64	152 (21.3)	51(19.9)	101 (22.1)
	65+	73 (10.3)	35(13.7)	38 (8.3)
	Total	712 (100)	256(100)	456 (100)

A total 256 (27 species) bacterial isolates belonging to 14 genera were recovered, of which 175 (68.4%) of the isolates (15 species) were Gram-negative and 81 (31.6%) isolates (12 species) were Gram-positive bacteria. *E. coli* and *K. pneumoniae*, were the two predominant Gram-negative bacteria consisting of (52.7%) and 7% of the total isolates, respectively. *S. sapropyticus* and *E. faecalis* were the first and the second predominant Gram positive bacteria, respectively (Tables 2 and 3).

**Table 2:** Distribution and frequency of Gram negative bacterial isolates

Genus	Species	*n* (%) of the total isolates
*Escherichia*	*E. coli*	135 (52.7)
*Klebsiella*	*K. pneumonia*	18 (7.0)
	*K. oxytoca*	1 (0.4)
*Pseudomonas*	*P. aeruginosa*	3 (1.2)
	*P. fluorescens*	1 (0.4)
	*P. luteola*	2 (0.8)
*Moraxella*	*M. nonliquefiens*	3 (1.2)
*Citrobacter*	*C. diversus*	2 (0.8)
	*C. freundii*	1 (0.4)
*Acinetobacter*	*A.baumannii*	2 (0.8)
*Providencia*	*P. alcalifaciens*	1 (0.4)
	*P. rettgeri*	1 (0.4)
*Francisella*	*F. tularensis*	1 (0.4)
*Morganella*	*M. morganii*	1(0.4)
*Sphingomonas*	*S. paucimobilis*	3(1.2)
Total (10)	15	175 (68.4)

**Table 3:** Distribution and frequency of Gram positive bacterial isolates

Genus	Species	*n* (%) of the total isolates
*Staphylococcus*	*S. saprophytics*	18 (7.0)
	*S. aureus*	9 (3.5)
	*S. warneri*	9 (3.5)
	*S. epidermidis*	8 (3.1)
	*S. hominis*	6 (2.3)
	*S. lentus*	3 (1.2)
	*S. haemolyticus*	2 (0.8)
*Streptococcus*	*S. agalactiae*	8 (3.1)
	*S. porcinus*	1 (0.4)
*Enterococcus*	*E. faecalis*	14 (5.5)
	*E. gallinarum*	1 (0.4)
*Kocuria*	*K. kristinae*	2 (0.8)
*Total (4)*	*12*	81 (31.6)

The overall drug susceptibility profile of Gram-negative bacteria for the 19 antibacterial drugs tested is summarized in Table 4. Ampicillin had the highest overall resistance rate (78.3%) for Gram negative bacteria followed by trimethoprim/sulfamethoxazole (66.3) and tetracycline (62.3%). Gram-negative bacteria showed better sensitivity towards piperacillin/tazobactam combination, cefoxitin, gentamicin, and nitrofurantoin with the overall resistance rates of 17.7, 24, 25.7, and 29.1%, respectively. *E. coli*, the most frequently isolated bacterium, showed 77.8, 70.4 and 69.6% resistance rates to ampicillin, trimethoprim/sulfamethoxazole, and tetracycline, respectively. The least resistance rate (20%) of the bacterium was observed for nitrofurantoin. *K. pneumoniae*, the second most commonly isolated Gram- negative bacterium exhibited a resistance rate of 100% for ampicillin and 66.7% for trimethoprim/sulfamethoxazole. The least resistance rate (5.6%) of this bacterium was observed for piperacillin/tazobactam combination. *Moraxella nonliquefiens* and *P. aeruginosa* the 3rd most frequently isolated Gram- negative bacteria were 100% resistant to 15 and nine drugs, respectively. *Acinetobacter baumannii* the 4th most frequently isolated bacteria was 100% resistant to ten drugs. Out of 175 isolates of Gram-negative bacteria, 2 (1.14%) isolates were resistant to all the antibiotics tested and 15 (8.6%) of the isolates were pandrug-resistant to cephalosporins.

**Table 4:** Percentage antimicrobial resistance profile of Gram-negative bacterial isolates (*n* = 175)

Species	Antibacterial drugs																		
	AM	AMC	CF	CZ	CXM	CXMA	FOX	CPD	CAZ	CRO	FEP	CIP	GM	LEV	FT	TM	TE	SXT	TZP
*E. Coli (135)*	77.8	45.2	59.3	42.2	43.7	45.2	22.9	37.8	35.6	34.8	43.7	50.4	28.1	55.6	20.0	39.3	69.6	70.4	21.5
*K. pneumoniae (18).*	100	22.2	55.6	50	44.4	44.4	5.6	44.4	44.4	44.4	50	16.7	22.2	11.1	61.1	38.9	44.4	66.7	5.6
*P. aeruginosa(3)*	100	100	100	100	100	100	100	100	33.3	100	33.3	33.3	0	0	100	0	100	100	33.3
*M. nonliquefaciensm(3)*	100	0	100	100	100	100	0	100	0	100	100	100	100	100	100	100	100	100	0
*C. diversus (2)*	100	100	100	100	50	100	100	50	50	50	50	50	0	0	0	0	0	100	0
*A.baumannii (2)*	100	100	100	100	100	100	100	100	0	100	0	50	0	0	100	0	0	0	0
*P. luteola (2)*	50	0	50	50	50	50	50	50	0	0	0	0	0	0	50	0	0	0	0
*K. oxytoca (1)*	100	0	0	0	0	0	0	0	0	0	0	0	0	0	100	0	0	0	0
*P.fluoresces (1)*	100	100	0	0	0	0	0	0	0	0	0	0	0	0	0	0	100	0	0
*M. morganii (1)*	100	100	100	100	100	100	100	100	100	0	0	100	0	0	100	0	0	100	0
*C. freundii (1)*	0	100	100	100	100	100	100	100	0	0	0	100	0	100	100	0	100	100	0
*P.alcalifaciens (1)*	0	0	0	0	0	0	0	0	0	0	0	0	0	0	100	0	0	0	0
*P.rettgeri (1)*	0	0	0	0	100	0	0	0	0	100	0	100	0	0	0	0	0	0	0
*S.Paucimobilis (3)*	0	0	0	0	0	0	0	0	0	0	0	0	0	0	0	0	0	0	0
*F. tularensis (1)*	0	0	0	0	0	0	0	0	0	0	0	0	0	0	0	0	0	0	0
All isolates	78.3	42.9	58.9	45.1	45.7	46.9	24	40.6	33.7	37.1	41.7	45.7	25.7	46.3	29.1	36	62.9	66.9	17.7

*AM* ampicillin, *AMC* amoxicillin/clavulanic acid, CF cefalotin, *CZ* cefazolin, *FEP* cefepime, *FOX* cefoxitin, *CPD* cefpodoxime, *CAZ* ceftazidime, *CRO* ceftriaxone, *CXM* cefuroxime, *CXMA* cefroxime axetil, *CIP* ciprofloxacin, *GM* gentamicin, *LEV* levofloxacin, *FT* nitrofurantoin, *TZP* piperacillin/tazobactam, *TE* tetracycline, *TM* tobramycin, *SXT* trimethoprim/sulfamethoxazole, *S* sensitive, *R* resistance, *P* pattern

The most common bacterial isolates found to be pandrug-resistant to cephalosporins were *E. coli, K. pneumoniae,* and *P. aeruginosa.*

Table 5 summarizes, the overall drug resistant pattern of Gram-positive bacteria for a panel of 16 antibacterial drugs tested. The highest overall resistance rate of Gram-positive bacteria was observed for erythromycin (82.2%), followed by tetracycline (75.6%) and clindamycin (68.4%) but, all Gram-positive bacterial isolates showed better sensitivity towards vancomycine with a sensitivity rate of 100% followed by daptomycin (98.1%), nitrofurantoin (97.1%), gentamicin (93%), and linezolid (92.1%). *S. saprophyticus*, the most frequently isolated Gram-positive bacterium, was 100% sensitive to vancomycine, minocycline and tigocycline. As depicted in Table 5, *E. faecalis,* the 2nd most frequently isolated Gram-positive bacterium was 100% susceptible to seven drugs.

**Table 5:** Percentage antimicrobial resistance profile of Gram-positive bacterial isolate (*n* = 81)

Species	Antibacterial drugs															
	CIP	CM	E	GM	LEV	MNO	MXF	FT	QDA	RA	TE	TGC	SXT	LIN	VA	DAP
*E. faecalis (14)*	7.1	7.1	85.8	0	0	85.8	0	0	100	0	78.6	7.1	7.1	7.1	0	0
*S. aureus (9)*	33.3	66.7	66.7	22.2	22.2	11.1	0	0	22.2	33.3	66.7	33.3	55.6	0	0	0
*S. epidermidis (8)*	62.5	100	75	0	62.5	12.5	0	0	50	50	62.5	25	25	37.5	0	–
*S.saprophyticus (18)*	27.8	88.9	94.4	16.7	38.9	0	38.9	5.6	38.9	50	72.2	0	55.6	5.6	0	5.6
*S. agalactiae (8)*	0	100	–	–	0	–	–	–	0	–	100	25	–	0	0	–
*S. haemolyticus (2)*	0	50	0	0	0	0	0	0	0	50	0	0	0	–	0	0
*S. lentus (3)*	100	33.3	66.7	0	66.7	66.7	33.3	0	66.7	33.3	100	0	100	0	0	0
*S. hominis (6)*	66.7	100	100	0	66.7	50	0	0	66.7	66.7	83.3	0	50	0	0	0
*S. warneri (9)*	44.4	66.7	77.8	0	22.2	22.2	11.1	11.1	11.1	44.4	77.8	0	55.6	0	0	–
*K. kristinae (2)*	100	50	100	0	–	–	–	–	–	–	–	–	–	–	–	–
S. porcinus (1)	0	–	100		–	–	–	–	–	–	–	–	–	–	–	–
E. gallinarum (1)	100	–	100	–	100	100	–	0	–	–	100	–	–	100	0	–
All isolates	34.6	68.4	82.2	7.0	29.5	31.4	13.1	2.9	44.2	37.7	75.6	10.4	42	7.9	0	1.9

*CIP* ciprofloxacin, *CM* clindamycin, *E* erythromycin, *GM* gentamicin, *LEV* levofloxacin, *MN0* minocycline, *MXF* moxifloxacin, *FT* nitrofurantoin, *QDA* ouinupristin/dalfopristin, *RA* rifampicin, *TE* tetracycline, *TGC* tigecycline, *LIN* linezolid, *SXT* trimethoprim/sulfamethoxazole, *VA* vancomycin, *DAP* daptomycin, *S* sensitive, *R* resistance

P = Pattern

*MIC of K. kristinae, S. porcinus and S. agalactiae was carried out by disc diffusion assay method*

*- = Not tested*

## Discussion

Urinary tract infection is caused by both Gram-negative and Gram-positive bacteria. However, the most commonly encountered bacteria are Gram negative in which *E. coli* consisting of the largest proportion of bacterial uropathogen worldwide [7, 17]. This is evident by the present study in which, out of 256 (27 species) bacterial isolates recovered, 175 (68.4%) were Gram- negative bacteria. Our finding of Gram-negative bacteria as the predominant species in patients with UTI was consistent with similar studies conducted locally [18–23] and internationally [7]. In the present study, 77.1% of the Gram negative bacterial isolates and 52% of the total bacterial isolates were strains of *E. coli. E. coli* has been reported as the main bacterial uropathogen accounting for 75 to 90% of bacterial isolates among patients with UTI [24, 25]. *E. coli* as the predominant bacterial uropathogen in the present study was consistent with similar studies conducted locally [18–23]. The prevalence of other predictable bacterial uropathogens varies from region to regions and from one study to another study [26, 27]. In this study, *S. saprophyticus and K. pneumonia* were the 2nd predominate isolates each consisting of 7% of the total bacterial isolates.

Of the 712 clinical samples collected from patients with cases of UTI, bacteria were isolated in 256 (36%) clinical samples. Urinary tract infection in the present study was relatively higher than similar local studies [18–23]. Local studies reported UTI in the range of 9 to 22.7%. Disparity in the rates of UTI in different studies could result from difference in the definition of bacteriuria, methodology, the length of the study period, size and type of study population.

In our study, the majority of UTI was recorded from females indicating that women are more likely to develop UTI than men. Our result, in this regard was in concordance with the findings of similar studies [28–31]. Women are more prone to develop UTI than men probably due to their anatomical and physiological changes [28, 32, 33]. Age groups of 25–44 and 45–64 were more affected with the infection than other age groups. Our finding in this regard was in agreement with the results of a studies done locally [23] and internationally [34].

In the current study, drug susceptibility testing of Gram negative and Gram- positive bacteria was performed against a panel of 19 and 16 antibacterial drugs, respectively. The number of drugs tested against urinary isolates in the present study was far greater than the number of drugs tested in previous studies in Ethiopia (18–23] and this may increase the option for the selection of drugs for the treatment of urinary tract infections.

The overall drug resistance rates of the Gram-negative bacterial isolates ranged from 17.7% for piperacillin/tazobactam combination and 78.3% for ampicillin. Lower resistance rates of Gram- negative bacteria for ampicillin than our study have been reported in studies conducted in Italy (36%) [35], UK (23%) [36], USA (43%) [37], Canada (33%) [11] and Norway (25%) [38]. However, a resistance rate of 87%, which is higher than ours, has been reported in India [39]. The resistance rates of bacterial uropathogens for ampicillin have also been found out to be 45, 50 and 100% in children from Canada, Europe and Africa, respectively [40–42]. High resistance rates of bacterial uropathogens for trimethoprim/sulfamethoxazole combination (66.3), in which the first choice of drug for the empirical treatment of UTI in Ethiopia and tetracycline (62.3%) was also observed in the present study. Our result was concurrent with similar study conducted in Ethiopia [23]. A notable observation was that the majority of Gram negative bacteria showed higher sensitivity pattern towards nitrofurantoin, gentamicin, cefoxitin and piperacillin/tazobactam with a sensitivity of 70.1, 74.3, 76 and 82.3%, respectively. As far as species specific antimicrobial resistance rates are concerned, *E. coli* the first more frequently isolated bacterium, showed high level of resistance (70–79%) for trimethoprim/sulfamethoxazole and ampicillin respectively. Similarly, *K. pneumoniae* the 2nd most frequently isolated Gram- negative bacterium demonstrated high level of resistance (66.7–100%) for trimethoprim/sulfamethoxazole and ampicillin, respectively. Similar result was obtained in a study conducted by Lu et al. [43, 44]. However, *E. coli* and *K. pneumoniae* revealed low level of resistance for nitrofurantoin (20%) and piperacillin/tazobactam (5.6%), respectively.

The overall drug resistance rates of Gram-positive bacterial isolates ranged from 0% for vancomycine and 82.2% for erythromycin followed by tetracycline (75.6%) and clindamycin (68.4%). An overall resistance rate of 85.6 and 76.7% of uropathognes for erythromycin and tetracycline, respectively has been reported in a similar study conducted in Ethiopia [23]. However, the majority of Gram-positive bacterial isolates showed higher sensitivity pattern towards vancomycine, daptomycin, nitrofurantoin, gentamicin, and linezolid with a sensitivity rates of 100, 98.1, 97.1, 93.0 and 92.1% respectively. As far as species specific antimicrobial resistance rates are concerned, *S. saprophytics,* the first more frequently isolated Gram-positive bacterium, showed high level of resistance (88.9–94.4%) for clindamycin and erythromycin, respectively. However, the bacterium was 100% sensitive to vancomycine, minocycline, tigocycline and 94.4% to linozilid and daptomycin. Contrary to our finding, a study conducted in Ethiopia by Amare et al. [45] documented 4.5% prevalence rate of vancomycin resistant Coagulase -Negative Staphylococci.

Our result revealed that Gram-positive and Gram-negative bacteria isolated in this study were more resistant to the commonly prescribed drugs in Ethiopia such as erythromycin, tetracycline, clindamycin, ampicillin, and trimethoprim/sulfamethoxazole combinations than of the drugs tested. The reason for this might be an irrational usage, easy availability and the over the counter sale of the antimicrobials without a proper prescription and an appropriate dosing schedule.

## Conclusion

Observation of many bacterial species implicated in causing UTI in this study warrants, a continuous epidemiological survey of UTI in health institutions across the country. High level of drug resistance against the commonly prescribed drugs necessitates a search for other options.

### Limitation of the study

Lack of clinical information to confirm whether urinary tract infections were hospital or community-acquired and complicated or uncomplicated were the major limitations of the present study.
